# Limited Sampling Modeling for Estimation of Phenotypic Metrics for CYP Enzymes and the ABCB1 Transporter Using a Cocktail Approach

**DOI:** 10.3389/fphar.2020.00022

**Published:** 2020-02-14

**Authors:** Eduardo Barbosa Coelho, Diego Alberto Ciscato Cusinato, João Paulo Ximenez, Vera Lucia Lanchote, Claudio José Struchiner, Guilherme Suarez-Kurtz

**Affiliations:** ^1^ Faculdade de Medicina de Ribeirão Preto, Universidade de São Paulo, Ribeirão Preto, Brazil; ^2^ Faculdade de Ciências Farmacêuticas de Ribeirão Preto, Universidade de São Paulo, Ribeirão Preto, Brazil; ^3^ Escola de Matemática Aplicada Fundação Getúlio Vargas, Rio de Janeiro, Brazil; ^4^ Coordenação de Pesquisa Instituto Nacional de Câncer, Rio de Janeiro, Brazil

**Keywords:** caffeine, fexofenadine, midazolam, metoprolol, omeprazole

## Abstract

Plasma concentration data points (n = 2,640) from 16 healthy adults were used to develop and validate limited sampling strategies (LSS) for estimation of phenotypic metrics for CYP enzymes and the ABCB1 transporter, using a cocktail of subtherapeutic doses of the selective probes caffeine (CYP1A2), metoprolol (CYP2D6), midazolam (CYP3A), losartan (CYP2C9), omeprazole (CYP2C19), and fexofenadine (ABCB1). All-subsets linear regression modelling was applied to estimate the AUC_0–12h_ for caffeine, fexofenadine, and midazolam, and the AUC_0–12h_ ratio of metoprolol: α-OH metoprolol and omeprazole:5-OH omeprazole. LSS-derived metrics were compared with the parameters’ ‘best estimates’ obtained by non-compartmental analysis using all plasma concentration data points. The correlation coefficient (*R* ^2^) was used to identify the LSS equations that provided the best fit for *n* timed plasma samples, and the jack-knife statistics was used as an additional validation procedure for the LSS models. Single time-point LSS models provided *R* ^2^ values greater than 0.95 (*R* ^2^ > 0.95) for the AUC_0–12h_ ratio of metoprolol:α-OH metoprolol and omeprazole:5-OH omeprazole, whereas 2 time-point models were required for *R* ^2^ > 0.95 for the AUC_0–12h_ of caffeine, fexofenadine, and midazolam. Increasing the number of sampling points to three led to minor increases in *R* ^2^ and/or the bias or prediction of the estimates. In conclusion, the LSS models provided accurate prediction of phenotypic indices for CYP1A2, CYP2C19, CYP2D6, CYP3A, and ABCB1, when using subtherapeutic doses of selective probes for these enzymes and transporter.

## Introduction

Phenotyping for drug metabolizing enzymes and transporters using simultaneous administration of selective substrates is frequently adopted in drug interaction studies. By appropriate choice of drug probes, phenotypic metrics, and sampling times, this “cocktail” approach enables concomitant assessment of the activity of drug metabolizing enzymes and transporters with economy of scale, both in terms of costs and time. Several cocktails have been developed since the pioneer work of Schellens et al. (1988), and comprehensively reviewed by different authors (Fuhr et al., 2007; de Andres and Llerena, 2016; Fuhr et al., 2019). A common feature of many cocktails is the use of single timed blood and/or urine sample(s) for quantification of the probe drugs and/or their metabolite(s), and inference of pharmacokinetic parameters to be used as phenotypic indices. The present article examines the impact of increasing the number of sampling time-points on the accuracy of phenotypic metrics for cytochrome P450 (CYP) enzymes and the ABCB1 transporter. We applied limited sampling strategy (LSS) modeling to plasma concentration data from a trial in which a cocktail of subtherapeutic doses of selective probes was used to investigate the effects of a Brazilian propolis extract on the metabolic activity of CYP enzymes (CYP1A2, CYP2C9, CYP2C19, and CY2D6) and the drug transporter ABCB1 (Cusinato et al., 2019a). Propolis is a resinous balsamic material, with a complex chemical composition, that is collected by bees from sprouts, exudates of trees, and other parts of the plant sand mixed with wax and bee enzyme (Sforcin and Bankova, 2011). The formulation used, which consists predominantly of green propolis from *Baccharis dracuncufolia*, was reported to have no clinically-relevant effect on the activity of the CYP enzymes examined or the ABCB1 drug transporter (Cusinato et al., 2019a). The results of the present study show that single-point LSS models for metoprolol and omeprazole provide highly accurate estimates of the metabolic activity of CYP2C19 and CYP2D6, but 2-point LSS models are required to obtain comparable accuracy when caffeine, midazolam, or fexofenadine are used as probes for CYP1A2, CYP3A, and the drug transporter, ABCB1, respectively.

## Methods

### Study Protocol

Details of the original trial, which was approved by the Ethics Committee of the Teaching Hospital of the Ribeirão Preto Medical School, University of São Paulo, and the effects of the propolis formulation have been published elsewhere (Cusinato et al., 2019a). Briefly, 16 healthy adults (11 men) ingested, on two occasions separated by 15 days, a capsule containing subtherapeutic doses of the phenotypic probes caffeine (10 mg; probe for CYP1A2), losartan (2 mg; CYP2C9), metoprolol (10 mg; CYP2D6), midazolam (0.2 mg; CYP3A), omeprazole (2 mg; CYP2C19), and fexofenadine (10 mg; ABCB1). During the 15 days between the two study phases, the subjects self-administered three oral daily doses of 125 mg of a standardized propolis formulation, namely EPP-AF^®^ (Apis Flora, Ribeirão Preto, SP, Brazil). Subjects were instructed to avoid caffeine-containing beverages in the five days preceding each study phase. In the two study phases, consecutive blood samples were collected immediately before (zero time) and 15, 30, 45, 60, 90, 120, 180, 240, 300, 360, 480, 600, and 720 min after ingestion of the phenotypic probes. The plasma concentrations of each probe and of metabolites of losartan (E-3174), metoprolol (α-OH metoprolol), and omeprazole (5-OH omeprazole) were quantified by validated LC/MS/MS methods (Cusinato et al., 2019b). The area under the plasma concentration *versus* time curve between 0 and 720 min (AUC_0–12h_) of the probes and metabolites was calculated using the trapezoidal method. The AUC_0–12h_ thus obtained is taken as the “best estimate” of the respective parameter value (Suarez-Kurtz et al., 1999; Suarez-Kurtz et al., 2001).

### Development of LSS Models

All-subsets linear regression analysis (Jodrell et al., 1996) was applied to the plasma drug concentration data sets from the first study phase (“training set”) for development of LSS models to predict the AUC_0–12h_ of caffeine, fexofenadine, midazolam, and the ratio of AUC_0–12h_s of metoprolol and omeprazole to their respective metabolites. We did not attempt to develop LSS models for losartan as a CYP2C9 probe, in view of the high plasma concentrations of the metabolite E-3174 in the last sample collected in both study phases. This suggested to us that the AUC_0–12h_ of E-3174 is not a reliable biomarker of CYP2C9-mediated conversion of losartan into E-3174.

A total of 1,320 plasma concentration data points were available for LSS development. In addition to the plasma concentration data, *CYP2C19* and *CYP2D6* genotypes and inferred phenotypes were available for all subjects enrolled in the trial (Cusinato et al., 2019a). One subject classified as CYP2D6 poor metabolizer (genotype CYP2D6*4/*4) was excluded from the LSS modeling for metoprolol:α-OH metoprolol, since the plasma concentration of the metabolite was below the limit of quantification in most samples. Two subjects identified as intermediate CYP2C19 metabolizers (genotype CYP2C19*1/*2) were included in the LSS modeling for omeprazole:5-OH omeprazole.

LSS models to predict the AUC_0–12h_ of caffeine, fexofenadine, and midazolam were derived using the respective drug plasma concentrations. In the case of metoprolol and omeprazole, the ratio of plasma concentrations of probe:metabolite at each sample time was used to develop LSS models to predict the ratio of the AUC_0–12h_ of probe:metabolite. Computations were performed using function leaps (Furnival and Wilson, 1974) in the R package software, version 3.5.0. This analysis produced linear equations of the following form: AUC_0–12h_ = *Ao* + *A*1*C1 + *A*2*C2…. + An* Cn, where *Ao* is an intercept, *An* are coefficients, and *Cn* are plasma concentration data at particular sampling times. Regression equations were ranked according to the correlation coefficient (*R* ^2^) criterium in order to identify those that provided the best fit for *n* timed plasma samples. The LSS-predicted AUC_0–12h_ of caffeine, midazolam, and fexofenadine, and LSS-predicted ratio of AUC_0–12h_s of metoprolol: α-OH metoprolol and omeprazole:5-OH omeprazole were then compared with the corresponding individual best estimates of the respective metrics. The bias of the LSS-derived estimates was assessed by calculating the mean percentage of difference (MD%) from the best estimates as follows: MD% = [(derived estimate - best estimate)/best estimate]*100%. Precision was assessed by calculating the mean absolute percentage of difference (MAD%) as follows: MAD% = [(|derived estimate - best estimate|)/best estimate]*100 (Suarez-Kurtz et al., 1999; Suarez-Kurtz et al., 2001). Linear correlation and Bland–Altman plots (Bland and Altman, 1986) were used to visualize the agreement between best-estimated and LSS-predicted metrics for each probe.

### Validation of the LSS Models

The LSS models were validated using two procedures: first, the LSS equations derived from the training set (first study phase) were used to estimate the corresponding AUC_0–12h_s or ratio of probe:metabolite AUC_0–12h_s using the concentrations observed at the same respective times in the second study phase (validation set; n = 1,320 samples). The AUC_0–12h_s or ratio of probe:metabolite AUC_0–12h_s thus obtained were then compared to the best estimates of the respective metric.

As a second validation approach, we used the jack-knife prediction (Ingram and Block, 1984) which is made by systematically leaving out one data set and using the *n-1* remaining data sets to develop LSS models with the same sampling times as the LSS equation that is being validated. Thus, *n* slightly different regression equations are obtained, and each of them is used to predict the AUC_0–12h_ or AUC_0–12h_ ratio for the data set that was left out. By discarding one data set at a time and fitting a new model for the *n* − 1 remaining data sets, the particular data set that is omitted does not influence the estimation of the regression parameters. The jack-knife validation was applied separately to the data sets of the two study phases. Linear correlation plots were constructed to visualize the agreement between best-estimated and LSS-predicted AUC_0–12h_ metrics using the jack-knife approach.

### Statistical Analysis

The specific statistical tests applied to the data are indicated in the text. Significance level was set at a *P* value < 0.05.

## Results

**Table 1** shows the AUC_0–12h_ of caffeine, fexofenadine, midazolam metoprolol, α-OH metoprolol, omeprazole, and 5-OH omeprazol in the two phases of the study.

**Table 1:** AUC_0–12h_ of the phenotypic probes and metabolites.

Probes and metabolites	Training set (ng∙h^−1^∙mL^−1^)	Validation set (ng∙h^−1^∙mL^−1^)
	Median (IQR)	Median (IQR)
Caffeine	1134.7 (627.6–1507.3)	1009.5 (584.0–1280.9)
Fexofenadine	59.8 (48.4–84.6)	54.6 (47.6–60.0)
Midazolam	1.1 (0.8–1.5)	1.3 (1.0–1.7)
Metoprolol	20.1 (10.5–47.4)	19.9 (10.8–50.8)
α-OH metoprolol	31.5 (21.6–34.3)	30.3 (25.0–34.9)
Omeprazole	20.7 (13.1–32.8)	18.3 (11.8–55.0)
5-OH omeprazole	55.6 (46.7–69.3)	55.7 (47.4–86.9)

### Development of LSS Models

**Figure 1:**
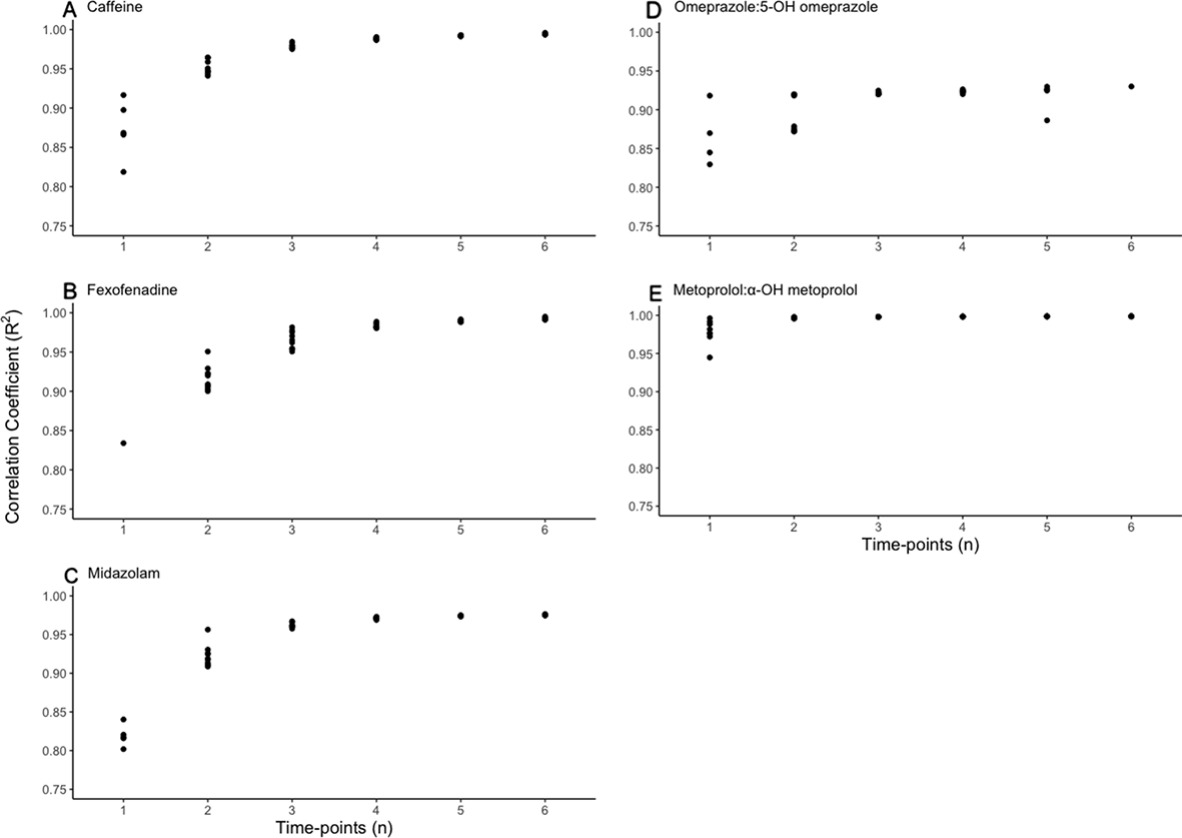
Scatter plots of the effect of increasing the number of sampling time-points (abscissa) on the correlation coefficient (*R* ^2^, ordinate) between best-estimated AUC_0–12_s **(A–C)** or AUC_0–12h_ ratios **(D, E)** and the corresponding LSS-predicted metrics.

The plasma concentration data sets from the first study phase (training set) were used for development of the LSS models. The results of these analyses (**Figure 1**) showed that single time-point LSS models provided *R* ^2^ values greater than 0.95 (*R* ^2^ > 0.95) for the AUC_0–12h_ ratios of metoprolol: α-OH metoprolol and omeprazole/5-OH omeprazole. For the AUC_0–12h_ of caffeine, fexofenadine, and midazolam, a minimum of 2 time-points were required for LSS models with *R* ^2^ > 0.95. Importantly, the sampling times for the best 1- or 2-point LSS models (i.e. those with the highest *R* ^2^ values) differed among the probes.

**Figure 2:**
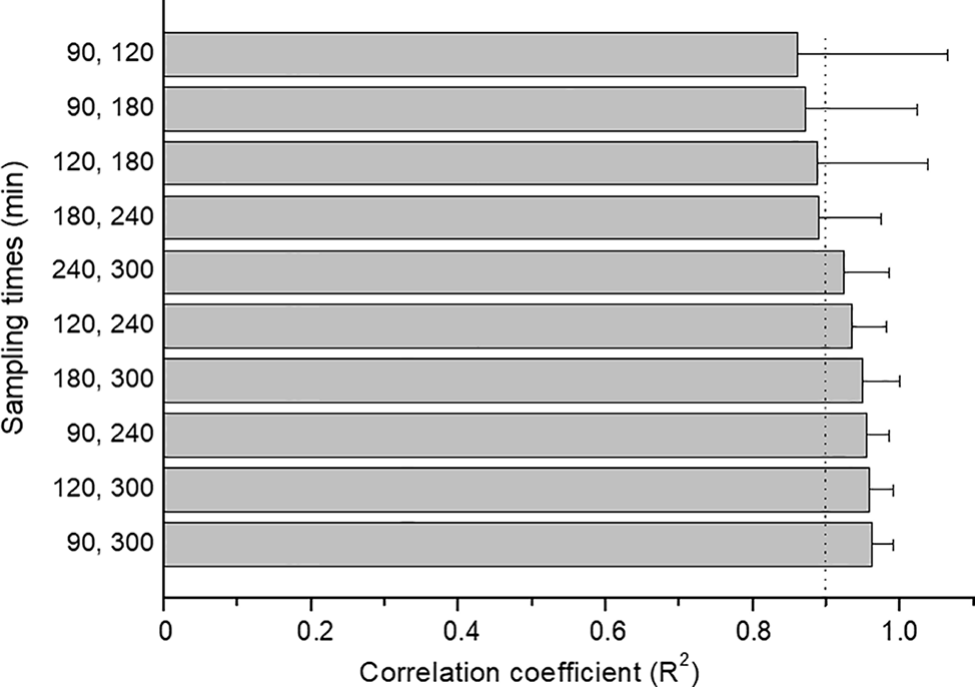
Bar plots of the mean ± S.D. of the *R* ^2^ values for the correlation between the best-estimated and the LSS-predicted AUCs of caffeine, fexofenadine and midazolam, and the AUC ratios of parent drug to metabolite for omeprazole and metoprolol, using different combinations of two sampling times. The dotted vertical line indicates *R* ^2^ = 0.95.

We contrasted these LSS models to identify the sampling times that provided the highest combined *R* ^2^ values for all five probes. This exercise excluded time-points (0, 15, 30, 45, 360–720 min) with plasma concentrations below the limits of detection for one or more probes or metabolites in one or more subjects. For one-time models, only the 120 min sample provided *R* ^2^ > 0.95 for both AUC_0–12h_ ratios of metoprolol: α-OH metoprolol and omeprazole/5-OH omeprazole, and the corresponding LSS models are presented in **Table 2**, with their regression equations, *R* ^2^, bias, and precision parameters. Four 2-point models provided average *R* ^2^ values greater than 0.95 for all five probes (**Figure 2**). There was no statistically significant difference in *R* ^2^ values among the four models (ANOVA, p = 0.67), and all models comprised either or both 240 and 300 min samples. Considering that a shorter time of sample collection would be more convenient, we selected for further analysis the 2-point models which did not comprise 300 min samples i.e. the models with paired samples collected at 90 and 120 min or 90 and 240 min. The corresponding *R* ^2^, bias, and precision parameters (**Table 2**), indicate that these 2-point LSS models provide accurate estimates of the AUC_0–12h_s of caffeine, fexofenadine, and midazolam, and the ratios of AUC_0–12h_s of metoprolol: α-OH metoprolol and omeprazole:5-OH omeprazole.

**Table 2:** *R* ^2^, bias, and precision of the LSS models.

Probe:metabolite sampling times (min)	LSS equations	*R* ^2^	MD% (mean ± SD)	MAD% (mean ± SD)
Caffeine				
90, 240	−59.306 + 3.030*C_90_ + 5.699*C_240_	0.93	1.48 ± 13.67	11.81 ± 6.37
120, 240	86.649 + 1.372*C_120_ + 7.018*C_240_	0.88	3.78 ± 16.04	12.99 ± 9.61
90, 120, 240	−64.496 + 2.969*C_90_ + 0.458*C_120_ + 5.193*C_240_	0.93	1.54 ± 13;63	11.49 ± 6.89
Fexofenadine				
90, 240	−5.830 + 2.614*C_90_ + 4.818*C_240_	0.96	0.35 ± 8.39	7.08 ± 4.13
120, 240	−2.789 + 2.076*C_120_ + 5.041*C_240_	0.90	1.59 ± 11.99	9.66 ± 6.85
90, 120, 240	−6.422 + 2.248*C_90_ + 0.454*C_120_ + 4.792*C_240_	0.96	0.29 ± 8.13	6.82 ± 4.07
Midazolam				
90, 240	−0.113 + 2.091*C_90_ + 6.445*C_240_	0.97	0.38 ± 10.91	8.56 ± 6.41
120, 240	−0.081 + 4.710*C_120_ + 0.720*C_240_	0.94	1.74 ± 13.31	11.51 ± 6.25
90, 120, 240	−0.114 + 2.069*C_90_ + 0.061*C_120_ + 6.365*C_240_	0.97	0.37 ± 10.86	8.54 ± 6.36
Metoprolol:α–OH metoprolol				
120	−0.026 + 0.7416*C_120_	0.98	−2.06 ± 12.33	9.57 ± 7.63
90, 240	0.024 + 0.063*C_90_ + 0.783*C_240_	0.99	−1.64 ± 13.49	9.55 ± 9.34
120, 240	0.006 + 0.296*C_120_ + 0.518*C_240_	1.00	−2.22 ± 9.27	6.39 ± 6.89
90, 120, 240	0.026 − 0.107*C_90_ + 0.394*C_120_ + 0.518*C_240_	1.00	−0.54 ± 7.45	5.26 ± 5.12
Omeprazole:5-OH omeprazole				
120	0.041 + 0.8880*C_120_	0.96	2.93 ± 16.46	16.57 ± 17.86
90, 240	0.100 + 0.272*C_90_ + 0.7499*C_240_	0.92	6.77 ± 28.62	20.11 ± 20.90
120, 240	0.044 + 0.849*C_120_ + 0.055*C_240_	0.96	3.13 ± 16.89	11.96 ± 11.96
90, 120, 240	0.021 − 0.655*C_90_ + 1.974*C_120_ − 0.401*C_240_	0.98	1.52 ± 12.15	9.48 ± 7.36

Increasing the number of sampling points to three (90, 120, and 240 min) led to relatively minor increases in *R* ^2^ and/or reductions in the bias or precision of the estimates (**Table 2**). Correlation plots of the best estimated *versus* the LSS-predicted AUC_0–12h_ metrics for each probe, and the respective Bland-Altman plots are shown in **Supplementary Figures 1–5**.

### Validation of the LSS Models

As a first validation procedure, the LSS equations derived from the training set, were applied to the data sets from the second study phase (validation set). The results shown in **Table 3** indicate that the single-point and 2-point LSS models derived from the development set provided accurate estimates of the respective metrics in the validation set, with *R* ^2^ values ranging from 0.89 to 0.98, except for midazolam, which showed lower *R* ^2^ values (0.73 and 0.81), but with bias and precision of estimates comparable to the other probes. Increasing the number of sampling points to three (90, 120, and 240 min) had no additional effect on the accuracy of the LSS for midazolam or the other probes.

**Table 3:** Validation the LSS models.

Probe:metabolite	*R* ^2^	MD% (mean ± SD)	MAD% (mean ± SD)
sampling times (min)			
Caffeine			
90, 240	0.95	3.60 ± 16.11	11.88 ± 11.08
120, 240	0.96	9.12 ± 11.53	11.39 ± 9.13
Fexofenadine			
90, 240	0.94	2.04 ± 11.29	8.69 ± 7.16
120, 240	0.88	5.44 ± 12.19	10.29 ± 8.20
Midazolam			
90, 240	0.81	5.63 ± 17.83	16.02 ± 8.83
120, 240	0.73	0.49 ± 22.62	14.67 ± 16.80
Metoprolol:α-OH metoprolol			
120	0.97	0.24 ± 12,98	11.42 ± 5.36
90, 240	0.97	5.12 ± 11.79	10.73 ± 6.60
120, 240	0.98	3.68 ± 10.42	8.40 ± 6.89
Omeprazole:5-OH omeprazole			
120	0.91	−4.48 ± 17.13	13.85 ± 10.51
90, 240	0.89	−0.14 ± 29.55	21.61 ± 19.36
120, 240	0.91	−4.20 ± 17.78	14.17 ± 11.00

**Figure 3:**
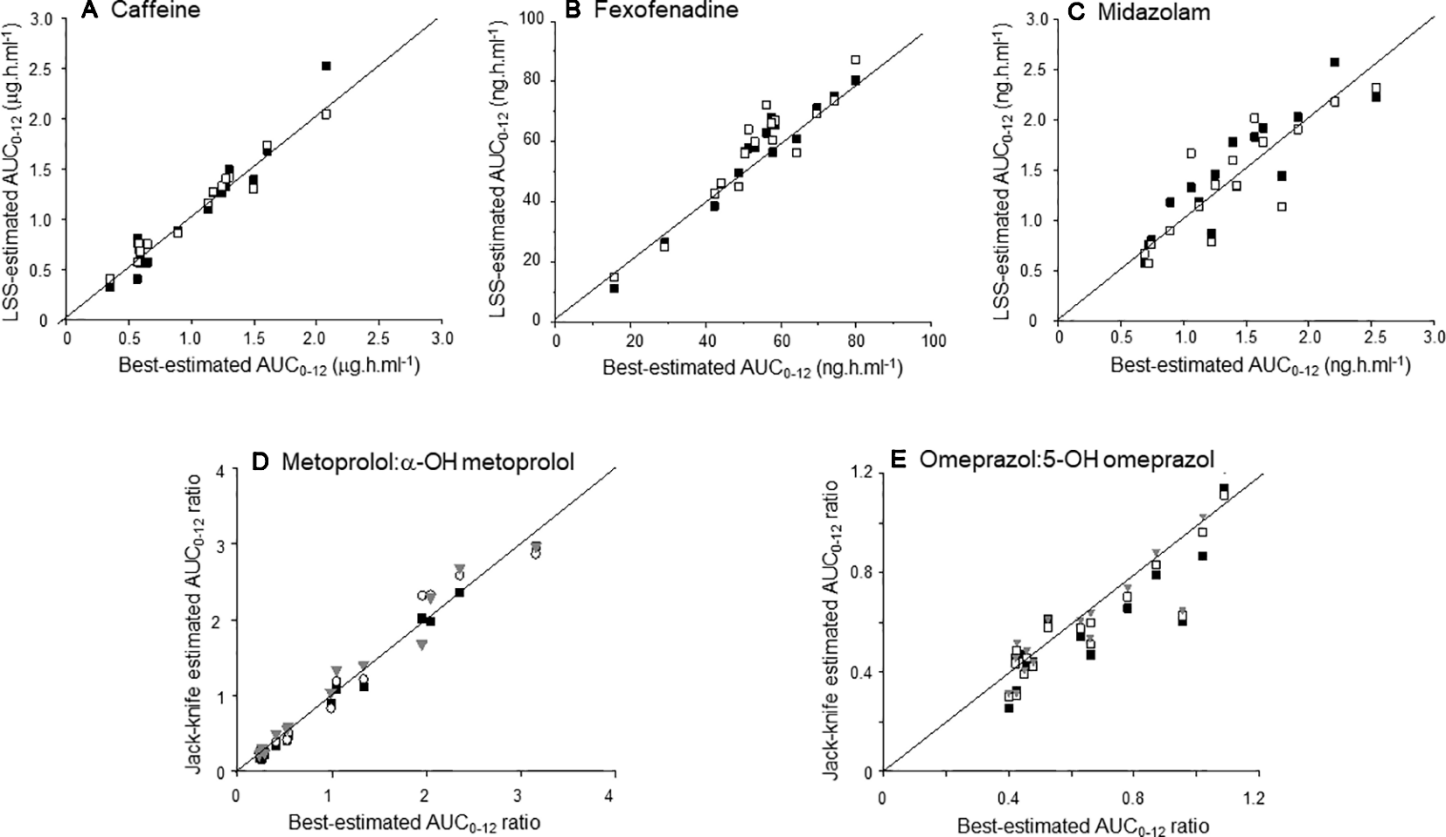
Scatter plot of the relationship between the best-estimated AUC_0–12_s **(A–C)** or AUC_0–12h_ ratios **(D–E)** and the corresponding LSS-derived metrics using the jack-knife approach to the validation data set. The LSS models are distinguished by symbols: single point models (90 min), grey triangles; two-point models, open squares (90 and 240 min), black squares (120 and 240 min). The continuous line in each plot is the identity line.

As a second validation approach, the jack-knife resampling technique was applied. Results obtained for the development and validation data sets are shown separately in **Table 4**, and diagnostic plots for the validation set are presented in **Figure 3**. *R* ^2^ values ranged from 0.73 (midazolam, 120 and 240 min, validation set) to 1.0 (metoprolol:α-OH metoprolol, two-point models, development set). Of notice, single-point models for metoprolol and omeprazole provided *R* ^2^ values > 0.89 for both datasets.

**Table 4:** *R* ^2^, bias (MD%), and precision (MAD%) for the jack-knife validation of LSS models.

Probe:metabolite sampling times (min)	Training set	Validation set
	*R* ^2^ (MD%, MAD%)	*R* ^2^ (MD%, MAD%)
Caffeine		
90, 240	0.93 (1.48, 11.81)	0.95 (3.60, 11.88)
120, 240	0.88 (3.78, 12.99)	0.96 (9.12, 11.39)
Fexofenadine		
90, 240	0.96 (0.35, 7.08)	0.94 (2.04, 8.69)
120, 240	0.90 (1.59, 9.66)	0.88 (5.44, 10.29)
Midazolam		
90, 240	0.97 (0.37, 8.55)	0.81 (5.63, 16.02)
120, 240	0.94 (1.73, 11.51)	0.73 (0.49, 14.67)
Omeprazole:5OH omeprazole		
120	0.92 (6.76, 20.10)	0.89 (−0.14, 21.61)
90, 240	0.96 (3.12, 11.94)	0.91 (−4.20, 14.17)
120, 240	0.98 (1.51, 9.46)	0.83 (−4.11, 17.14)
Metoprolol:α-OH metoprolol		
120	0.99 (−1.64, 8.96)	0.97 (5.12, 10.06)
90, 240	1.00 (−2.22, 6.00)	0.98 (3.68, 7.88)
120, 240	1.00 (−0.55, 4.93)	0.97 (6.55, 9.28)

## Discussion

In the present study, LSS strategies were applied to predict phenotypic metrics for a cocktail of probe drugs for CYP enzymes and the ABCB1 transporter. Plasma concentration data for the probe drugs and their metabolites in 2,640 samples collected from 16 closely monitored, healthy volunteers (Cusinato et al., 2019a) were used for development and subsequent validation of LSS models. All the probe drugs, namely caffeine (CYP1A2 probe), midazolam (CYP3A), omeprazole (CYP2C19), metoprolol (CYP2D6), and fexofenadine (ABCB1) have been previously included, albeit in higher doses, in phenotypic cocktails, notably the Basel (Donzelli et al., 2014) and the Geneva cocktails (Bosilkovska et al., 2014). The phenotypic indices used to assess the activity of the CYPs and ABCB1 transporter have been previously validated. For example, the Basel cocktail used the AUC ratios of parent drug to metabolite for omeprazole and metoprolol (Donzelli et al., 2014), the Geneva cocktail used the AUC of fexofenadine (Bosilkovska et al., 2014), the Inje cocktail adopted the AUC of midazolam (Ryu et al, 2007), and the caffeine AUC has been employed as a phenotypic metric for CYP1A2 activity in drug cocktail Interaction studies (Doroshyenko et al., 2013; Gazzaz et al., 2018).

The LSS modeling disclosed single time-points which provided highly accurate (*R* ^2^ > 0.95) estimates of the AUC_0–12h_ ratios for metoprolol: α-OH metoprolol and omeprazole:5-OH omeprazole. This supports the adoption of single point metabolic ratios for metoprolol (90, 120, or 240 min) and omeprazole (120 min) as reliable parameters of CYP2D6 and CYP2C19 activity, respectively. For the other drugs examined, namely caffeine, fexofenadine, and midazolam, 2-timed samples were required to obtain predictions of the respective phenotypic metrics with *R* ^2^ values exceeding 0.95. Although the most informative time points differed among these drug probes, LSS models based on paired samples collected at 90 and 120 min or 90 and 240 min provided *R* ^2^ values in the range of 0.88 to 1.0 for their respective phenotypic metrics in the development cohort (**Table 3**). Increasing the number of sampling points to three (90, 120, and 240 min) led to relatively minor increases in *R* ^2^ and/or reductions in the bias or precision of the estimates. The statistical principle of parsimony advises in favor of models with fewer parameters; accordingly, the two-point LSS models (90 and 120 min; 120 and 240 min) for all drug probes plus the single-point models (120 min) for metoprolol and omeprazole were chosen for independent validation. The two validation tests confirmed the robustness of the LSS models over wide ranges (> 5 fold) of phenotypic metrics values (**Figure 3**). We suggest that the validated LSS models are appropriate for predicting phenotypic indices for CYP1A2, CYP2C19, CYP2D6 CYP3A, and the ABCB1 transporter, using subtherapeutic doses of caffeine, omeprazole, metoprolol, midazolam, and fexofenadine, respectively.

Several phenotypic cocktails propose single-point plasma sampling for assessment of phenotypic indices for CYPs and ABCB1 (reviewed by Fuhr et al., 2007; de Andres and Llerena, 2016; Fuhr et al., 2019). The present results supports this approach when metoprolol and omeprazole are used, respectively, as phenotypic probes for CYP2D6 and CYP2C19 activity. However, including one additional sampling point increases the predictive power of phenotypic indices for caffeine, midazolam, and fexofenadine.

We acknowledge limitations of this study: first, CYP2D6 poor metabolizers were not included in the LSS modeling; thus, the single-point and two-point models for metoprolol may not be extended to individuals with this phenotype. Two other potential limitations are the relatively small size of our study cohort (n = 16) and the fact that it comprised only healthy subjects. However, these same limitations apply to the development of several other phenotyping cocktails, such as the Basel (n = 16; Donzelli et al., 2014), Inje (n = 12; Ryu et al, 2007), Cooperstown (n = 12; Streetman et al., 2000), Karolinska (n = 24; Christensen et al., 2013), Quebec (n = 10; Sharma et al., 2004), and the Ceiba cocktail (n = 13; de Andres et al., 2016). Of notice, among these phenotypic cocktails, only the CEIBA was developed in another Latin American population, namely Equatorians. Finally, our modeling is based on data from low, subtherapeutic doses of the probe drugs, and the phenotypic metrics analyzed may not have a linear relationship to the enzyme and transporter activities when higher probe doses are used.

## Data Availability Statement

The authors declare that all data supporting the findings of this study are available within the article and its **Supplementary Materials**, or from the corresponding author on request.

## Ethics Statement

All procedures performed in the studies involving human participants were in accordance with the ethical standards of the institutional and/or national research committee and with the 1964 Helsinki declaration and its later amendments or comparable ethical standards. Written informed consent was obtained from all subjects included in the study.

## Author Contributions

EC: responsible for research ethics application, data analyses, and reviewing the manuscript. DC: responsible for research ethics application, data collection, and analyses, and reviewing the manuscript. JX: responsible for development of limited sampling modeling and reviewing the manuscript. VL: responsible for data analyses and interpretation, and reviewing the manuscript. CS: responsible for development of limited sampling modeling and reviewing the manuscript. GS-K: responsible for study design, data analyses and interpretation, and writing the original manuscript.

## Funding

GS-K was a Visiting Professor at Faculdade de Ciências Farmacêuticas de Ribeirão Preto, sponsored by Universidade de São Paulo (USP). The authors acknowledge grant support from Conselho Nacional de Desenvolvimento Científico e Tecnológico (CNPq), Fundação de Amparo à Pesquisa do Estado de São Paulo (FAPESP), Fundação de Amparo à Pesquisa do Estado do Rio de Janeiro (FAPERJ), Fundação de Apoio à Assistência, Ensino e Pesquisa HCFMRP-USP (FAEPA), Departamento de Ciência e Tecnologia, Ministério da Saúde (DECIT/MS), and Financiadora de Estudos e Projetos (FINEP).

## Conflict of Interest

The authors declare that the research was conducted in the absence of any commercial or financial relationships that could be construed as a potential conflict of interest.
