# Supplementary material for: Limited Sampling Modeling for Estimation of Phenotypic Metrics for CYP Enzymes and the ABCB1 Transporter Using a Cocktail Approach
Source: Front Pharmacol. 2020 Feb 14;11:22. doi: 10.3389/fphar.2020.00022 (PMC7057125; doi:10.3389/fphar.2020.00022)

Supplementary Figures 1-5.

Supplementary Figures 1-5. Scatter (A) and Bland–Altman (B-D) plots of the LSS-derived *versus* the best-estimated pharmacokinetic metrics for caffeine ($AUC_{0\text{-}12h,}$ Figure 1), fexofenadine ($AUC_{0\text{-}12h,}$ Figure 2), midazolam ($AUC_{0\text{-}12h,}$ Figure 3), metoprolol (ratio of metoprolol:a-OH metoprolol $AUC_{0\text{-}12h}$´s, Figure 4) and omeprazole (ratio of omeprazolol:5-OH omeprazol $AUC_{0\text{-}12h}$´s, Figure 5). The abscissae in all panels refer to the best-estimated metrics. The ordinates represent the LSS-derived metrics in panels labeled A, and the difference between the best-estimated and LSS-derived metrics in the Bland-Altman plots (panels B - D). The continuous line in A is the identity line. The dashed lines in B – D are drawn at + 1.96 and -1.96 of the standard deviation of the mean difference between best-estimated and LSS-derived metrics. The symbols represent the different LSS models: black squares, 90 and 240 min; red circles, 120 and 240 min; green triangles, 90, 120 and 240 min.

Supplementary figure 1 - Caffeine

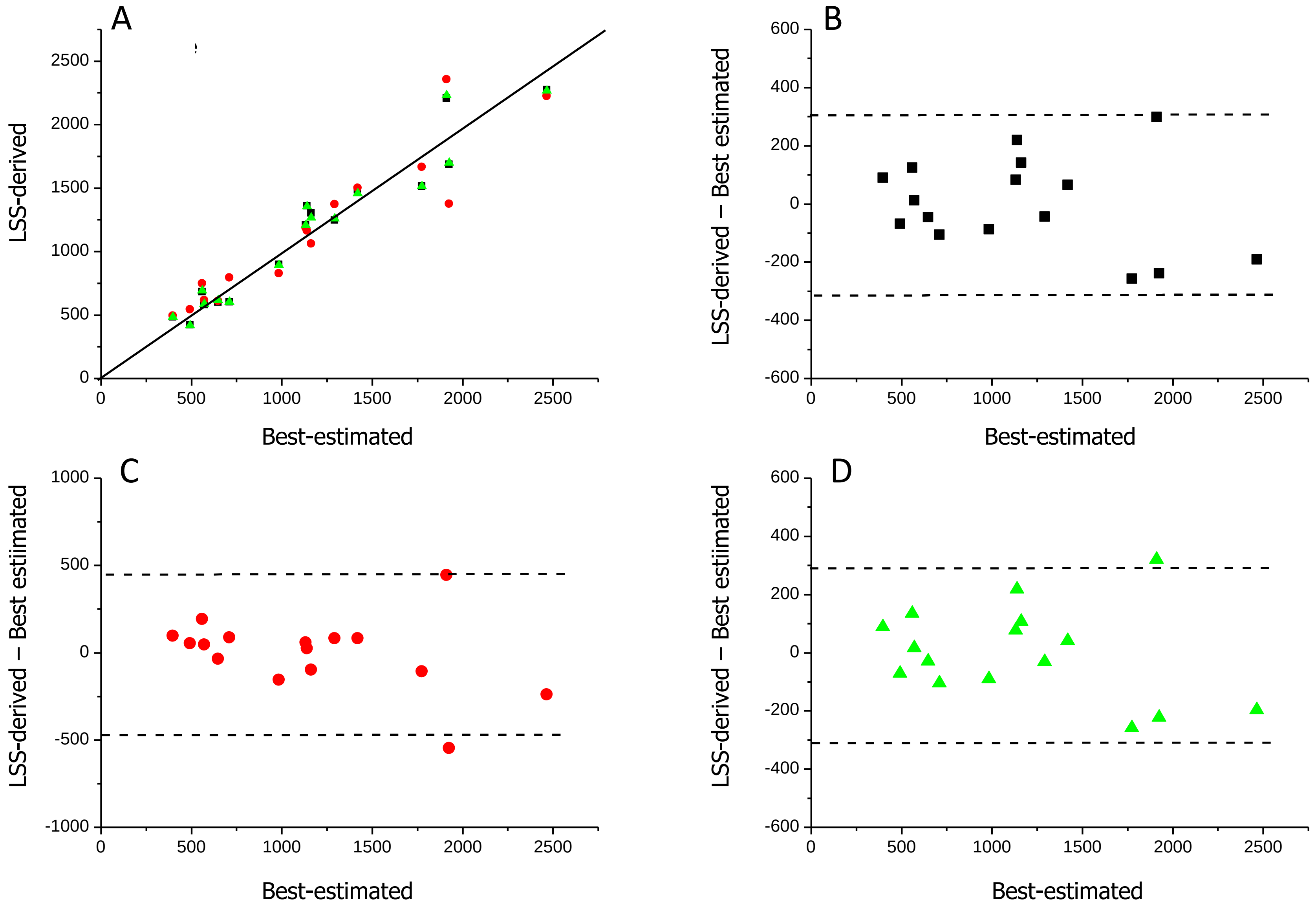

Supplementary figure 2 - Fexofenadine

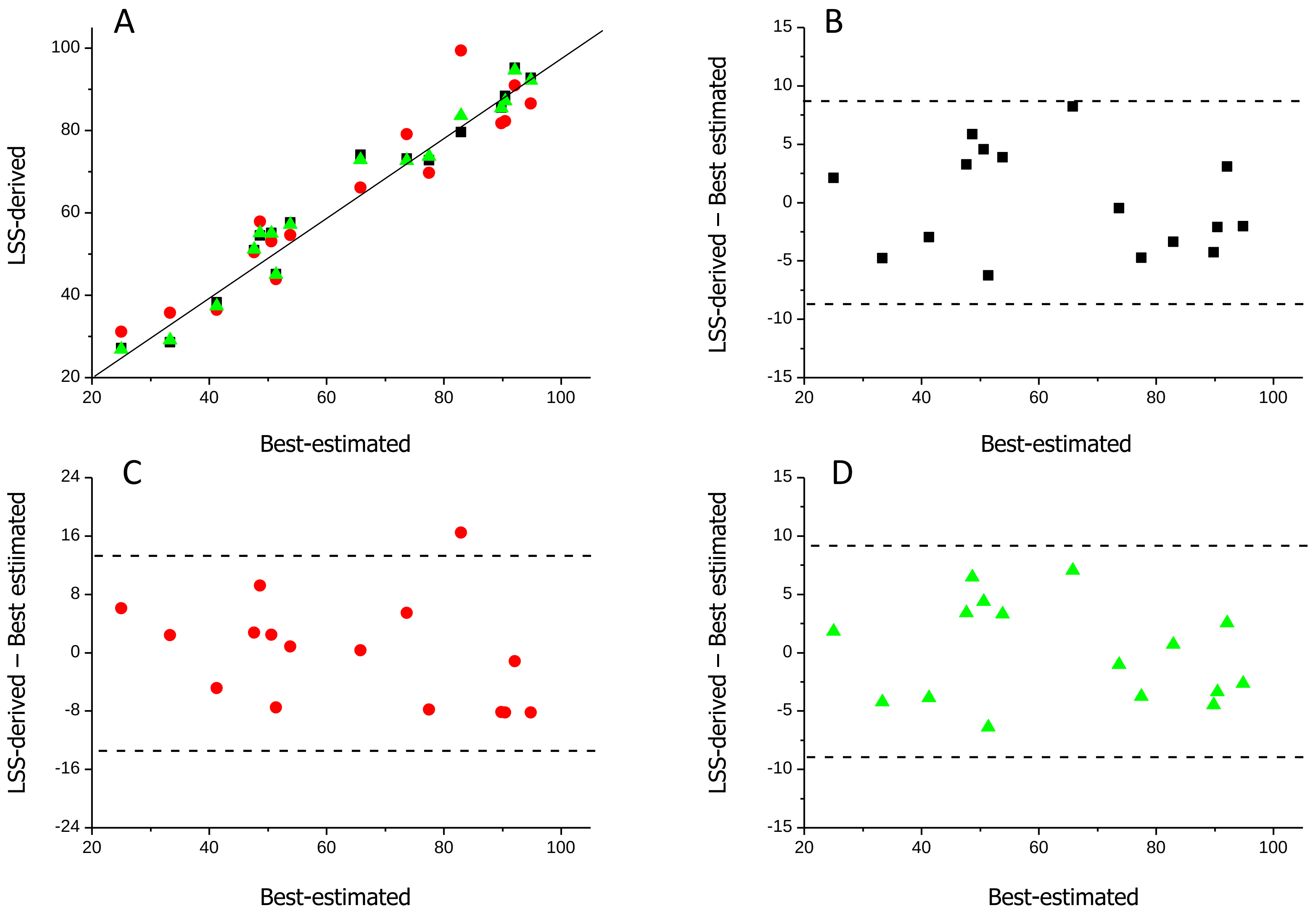

Supplementary figure 3 - Midazolam

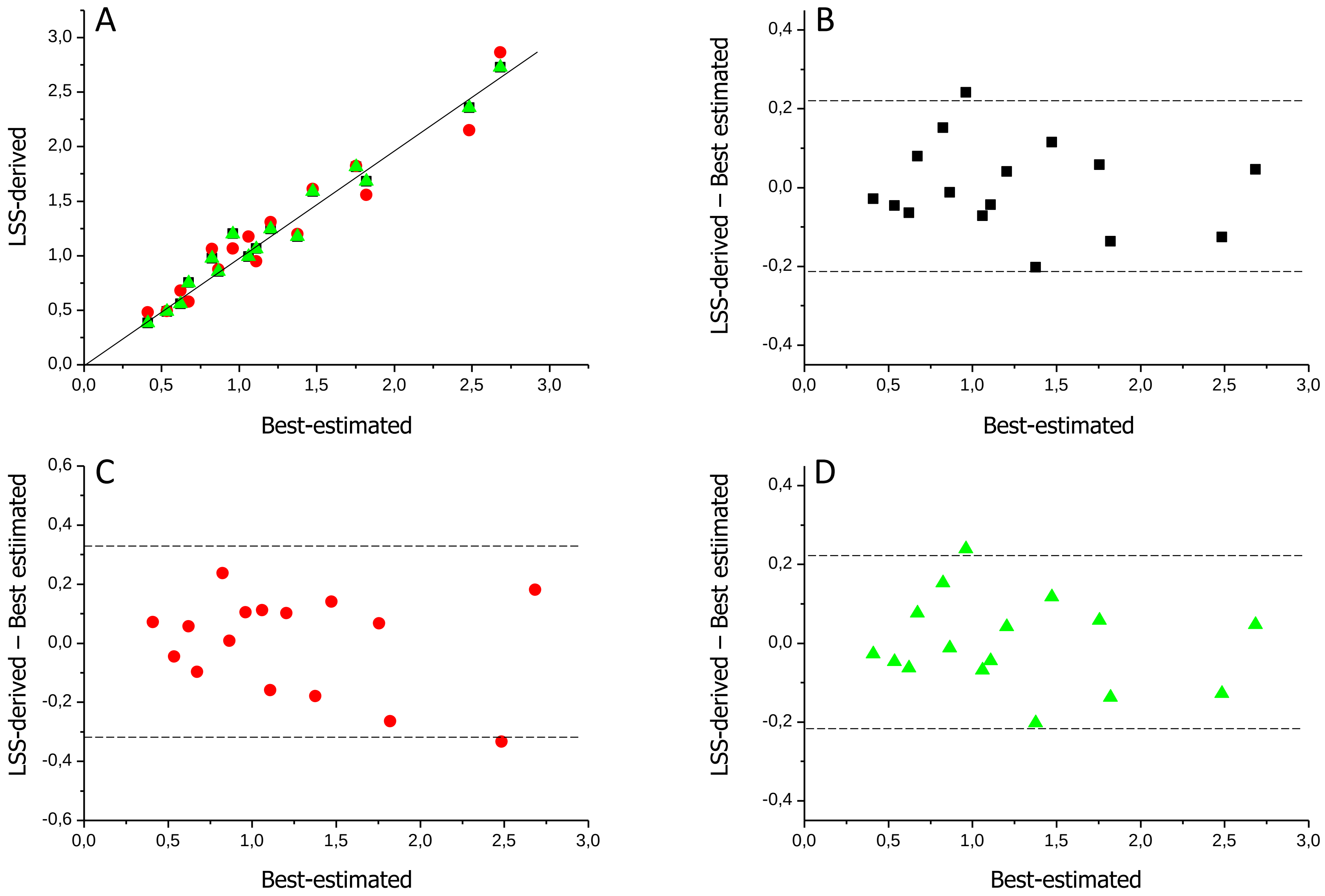

Supplementary figure 4 - Metoprolol:α-OH metoprolol

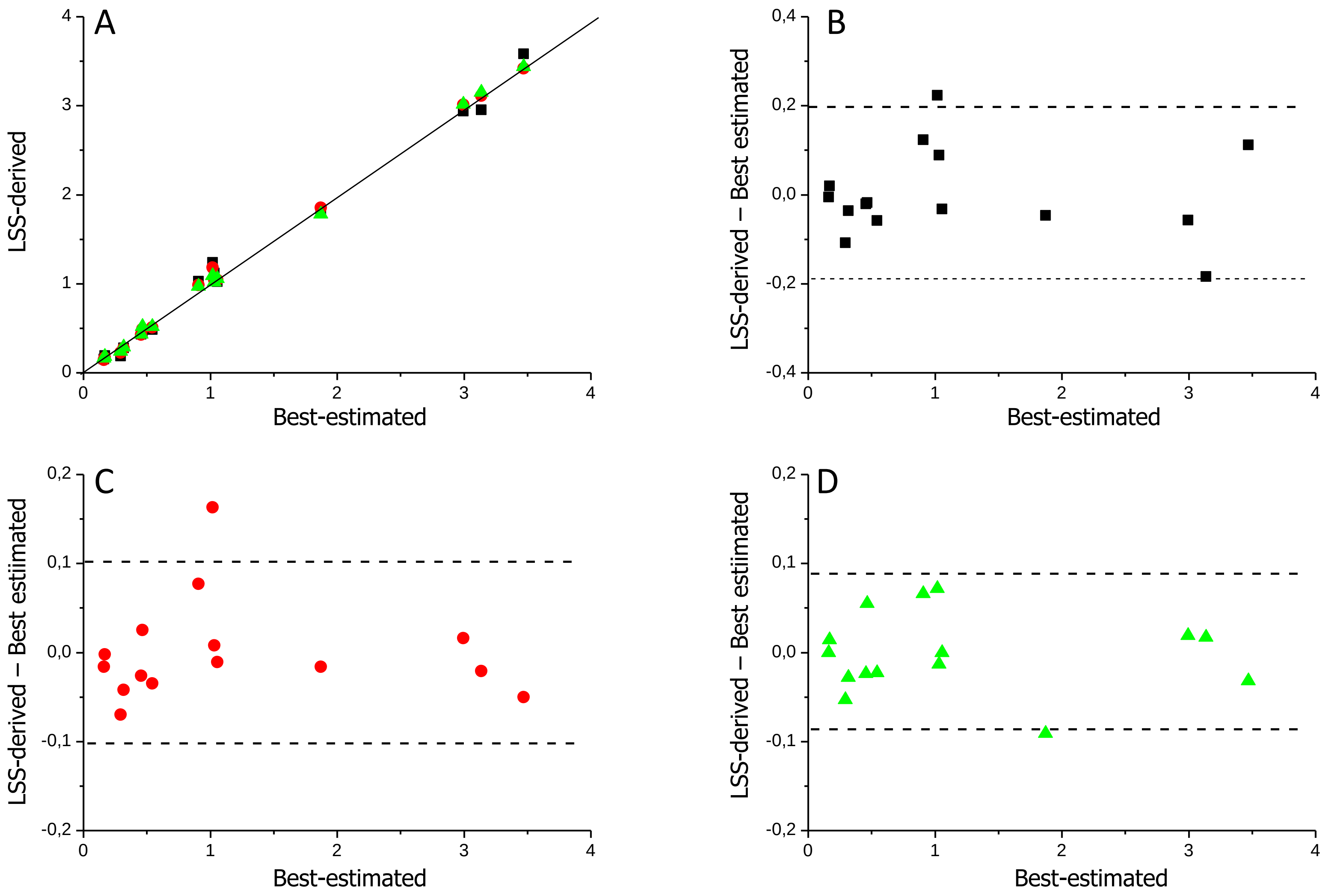

Supplementary figure 5 - Omeprazole:5-OH omeprazole

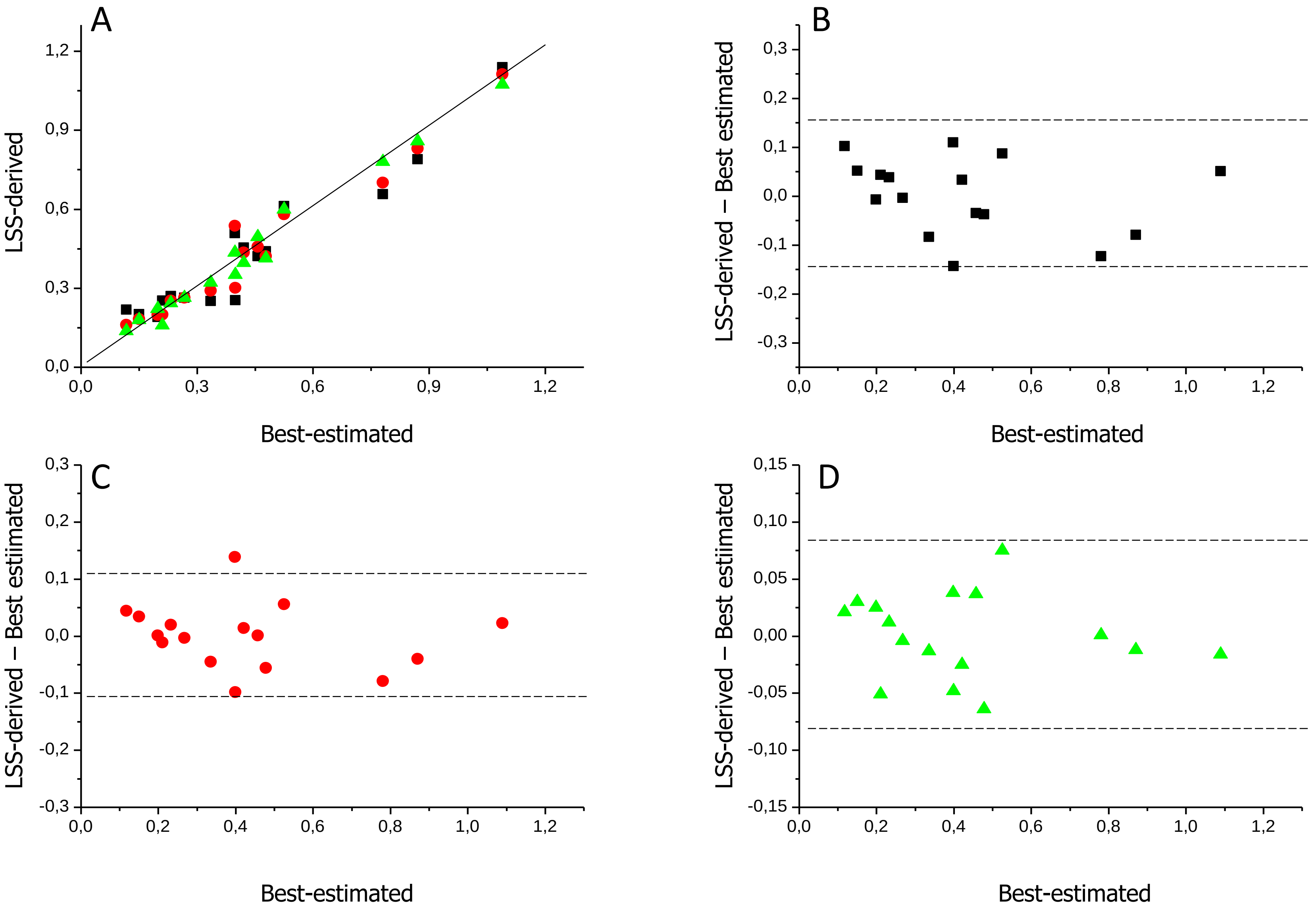

Supplement: Supplementary file 1 [file DataSheet_1.pdf]
